# Surrogate mother – praiseworthy or stigmatized: a qualitative study on perceptions of surrogacy in Assam, India

**DOI:** 10.1080/16549716.2017.1328890

**Published:** 2017-06-12

**Authors:** Anna Arvidsson, Polly Vauquline, Sara Johnsdotter, Birgitta Essén

**Affiliations:** ^a^ Department of Women’s and Children’s Health/IMCH, Uppsala University, Uppsala, Sweden; ^b^ Department of Women’s Studies, Gauhati University, Guwahati, India; ^c^ Faculty of Health and Society, Malmö University, Malmö, Sweden

**Keywords:** Childlessness, in-vitro fertilization (IVF) surrogacy, motherhood, surrogate mother, stigmatization, low-income setting

## Abstract

**Background**: Surrogacy is a reproductive practice that has been strongly marketed in India as a solution for childless couples. As a result, the number of surrogacy clinics is increasing. Meanwhile, a global discourse on surrogacy, originating from a Western perspective, has characterized surrogacy as being exploitative of women in low-income settings, where poverty drives them to become surrogate mothers.

**Objective**: This study explored perspectives on surrogacy from men and women in Assam, an Indian state known to be a low-income setting. Surrogacy arrangements in Assam are still uncommon. It can be expected that the dominant global discourses on surrogacy will be unfamiliar to the general population, and the objective was also to position the results within the divergent global discourses of surrogacy.

**Methods:** In order to explore local views on surrogacy, we conducted individual interviews and focus group discussions with people from various socioeconomic groups in Assam.

**Results**: Our findings reveal that people in Assam perceive surrogacy as a good option for a childless couple, as it would result in a child who is a ‘blood’ relation – something highly desirable for sociocultural reasons. However, the part played by the surrogate mother complicates local views on surrogacy. Most people consider payment to the surrogate mother contrary to societal norms. A surrogate mother is also often judged in a moral light, either as a ‘bad mother’ for selling her child, or as a ‘noble woman’ who has helped a childless couple and deserves payment for her services.

**Conclusions**: In order to decrease the stigmatization of women, a regulatory policy is needed that will take into account the complex understandings of surrogacy and perceptions of surrogate mothers in Indian society. In policy, the possible effect of the dominant exploitation discourse needs to be modulated by local understandings of this reproduction method.

## Background

Assisted reproductive technology (ART) is increasingly used by involuntarily childless couples all over the world. Surrogacy is one such ART that is both widespread and highly controversial for its involvement of a third party, the surrogate mother. The involvement of poor women as surrogate mothers has led to the emergence of two conflicting global discourses. The main discourse stems from the view that impoverished women become surrogate mothers out of desperation, and that their rights are disregarded when they assume that role. This view has resulted in allegations that such women are being exploited, especially in low-income settings [1–5]. A contrasting discourse describes surrogacy as an opportunity for the empowerment of poor women because it presents the possibility of gaining significant financial benefit as well as liberation from patriarchal control [6,7].

Transnational surrogacy typically takes place in areas where surrogate mothers can be found among the poor. India was once the primary country for transnational surrogacy, until December 2015, when the government instructed clinics not to accept new foreigners as clients.

Commercial in-vitro fertilization (IVF) surrogacy became legal in India in 2002, although it is poorly regulated by guidelines that were set in 2005 [3]. ART clinics promote their services (surrogacy being one of them) as a chance for Indian women to remedy a vulnerable childless situation [8]. Until recently, many of those using surrogacy in India were foreigners. However, reactions from both international and domestic organizations that have perceived surrogacy as exploitative have resulted in government pressure, effectively banning foreigners from surrogacy clinics, although there is no law to prohibit this practice [9].

The practice of surrogacy is rising among Indians themselves, although in many circles it appears to be taboo [10]. Studies of surrogate mothers have reported that they are often stigmatized because surrogacy is widely believed to involve sexual intercourse [11,12]. Public opinion about surrogacy, according to the available literature, has not been studied in India, while it has, to some extent, been studied elsewhere (e.g. in Greece [13], in the UK [14], in Australia [15,16], and in Sweden [17]). It can be expected that the dominant global discourses on surrogacy will be unfamiliar to the general population in India. Thus, when future policies are deliberated, insights into local understandings of surrogacy will be valuable. The aim of this study was to explore the perceptions of surrogacy and surrogate mothers in Assam, among men and women in different socioeconomic groups, in order to understand how this method of reproduction was compatible with cultural norms in the local context. The objective was also to position the results within the divergent global discourses of surrogacy.

## Methods

### Study setting

Assam is one of eight states in northeastern India. It shares international borders with Bhutan and Bangladesh. The population of Assam is 31 million; most inhabitants are Hindu (61%), and a large proportion are Muslim (34%) [18]. The majority (86%) live in rural areas [18] and most people are engaged in the agriculture sector [19]. Assam is considered to be one of the poorest states in India with a large proportion of marginalized groups [20]. The situation for women in Assam is, however, seen to be better in some respects than for those who reside in other parts of India. Women from Assam are believed to have more liberty and decision-making power at the household level than women elsewhere in the country [21,22].

Assam has had an increase in the number of private IVF clinics where reproductive issues are handled for those who can afford it, although such clinics are not widespread – especially not those providing surrogacy [23].

Assam, as our area of study, was purposely selected because surrogacy is not a very common phenomenon there, and this particular state would provide a unique opportunity for us to gather local opinions on surrogacy at an early stage in its introduction into society.

### Design and data collection

An exploratory prospective qualitative study was conducted between April 2012 and the end of 2013 in four periods of fieldwork lasting from two to seven weeks each. We started out by exploring the occurrence of surrogacy. In order to understand the extent to which IVF and surrogacy were available in the area, we interviewed four doctors at IVF clinics in the largest city of Assam: Guwahati. In addition, we collected information on reproductive health issues and surrogacy through informal talks with civil society organizations (CSOs). Subsequently, we conducted 27 semi-structured individual interviews and 15 focus group discussions (FGDs) with women and men ranging in age from 18 to 70 years. The interviews took place where it was convenient for the participants and afforded them some privacy: somebody’s home, an assembly room, a classroom, or in a private setting in a clinic or at the informants’ workplace. Data collection was facilitated by staff at primary health clinics in rural areas, who recruited participants from lower socioeconomic groups (mainly those who were unemployed, working in agriculture, working as weavers, or working as daily wagers). Co-workers at Gauhati University facilitated the recruitment through their contacts in villages, at colleges in rural and semi-urban areas, and at the University and the Rotary Club in Guwahati (Table 1). In the FGDs we strived to include informants from a variety of socioeconomic strata and a stratified sampling procedure was followed. The term socioeconomic here refers to level of education and income, but not caste, religion, or community (Table 1). It was not difficult to recruit informants; rather, more people than asked usually wanted to participate in the FGDs, and this was the case even though no compensation was offered for participating.

**Table 1.** Overview of material and data collection.

Level of socioeconomic background	Type of interview	Age	Area	Period
Lower	23 individual	20–38	Rural	Fall 2012
	(20 women, 3 men)			
Lower	7 FGD	20–70	Rural/urban	Fall 2013
	(4 with women, 3 with men)			
Students	5 FGD	18–23	Rural/semi-urban/urban	Fall 2013
	(2 with women, 2 with men, 1 mixed-sex)			
Middle	2 FGD	25–60	Semi-urban	Fall 2013
	(1 with women, 1 with men)			
Higher	1 FGD	43–60	Urban	Fall 2013
	(men)			
Higher	4 individual	38–49	Urban	Fall 2013
	(women)			

Note: FGD = Focus group discussion.

All interviews were carried out by the first author, in collaboration with a Swedish registered nurse midwife. All interviews were conducted in the informant’s native language or occasionally in English. A female interpreter, conversant with the local languages and well-informed on reproductive health issues, assisted during the interviews and FGDs.

Initially, semi-structured individual interviews were conducted with women and men from lower socioeconomic strata in a rural area. We chose to interview more women than men as we were at this point interested in women’s perspectives (Table 1). Questions were asked to determine what participants knew and thought about different solutions to childlessness, including surrogacy. When they did not know of surrogacy, it was explained by the interpreter as an IVF method where ‘the egg and sperm of a childless couple are put together, and then inserted inside the womb of a woman who will give birth to their child.’ The surrogate mother’s role was described as ‘a woman carrying a child for someone else, without using her own eggs.’ The possibility of involvement of money was explained subsequently.

The 15 FGDs were added as we wanted to explore how discussions about surrogacy and surrogate mothers were reflected upon in a larger group of informants from different socioeconomic groups [24]. FGDs were conducted with students, teachers (representing the middle socioeconomic group), and people in lower and higher socioeconomic groups. The FGDs were homogeneous with regard to social group, with the exception of one group, which turned out to have participants from a mixture of socioeconomic backgrounds. We have not specified the participants’ religion, although most were Hindus and only a few were Muslims or Christians, both among individual interviews and within the FGDs. There were 5 to 8 participants in each of the 15 FGDs, totaling 100 individuals.

In the FGDs, we wanted to gain their knowledge and perceptions in general on solutions to childlessness before probing about surrogacy, and a brief story, a vignette about a childless couple, was first presented by the interviewer. The group was then encouraged to discuss different solutions to the couple’s situation. Follow-up questions focused on perceptions of different solutions to childlessness, knowledge and perceptions on assisted reproductive technology, surrogacy, the surrogate mother, and the involvement of money in the surrogacy arrangement.

During FGDs, the first author explained the vignette and posed the follow-up questions, which were translated into the local language, often Assamese. During the discussions, the interpreter translated summaries of what was being said in order for the first author to adequately understand how the discussion unfolded. The translator, being well aware of the purpose of the study, also probed when necessary in order to deepen the discussion. Finally, four individual interviews with women from the higher socioeconomic group were conducted. In these interviews the same vignette about a childless couple was used with a similar method of probing to that used for the FGDs. All interviews and FGDs were audio recorded with the consent of the participants. All interviews and FGDs lasted from 30–60 minutes except for the final four interviews which lasted 45–90 minutes. The recordings were later transcribed and translated into English by a research assistant at the Department of Women’s Studies, Gauhati University, Assam. Some of the recordings were also listened to by the second author, who is familiar with the local languages, and back-translated to clarify the transcriptions. Also, further contextualization of the data was made possible through daily interaction with people in Assam, with whom we discussed surrogacy issues in informal ways.

### Data analysis

The study relies on a social constructivist approach, with the belief that there are multiple context-bound realities, and that definitions of identities or phenomena depend on the context [25].

Thematic analysis was conducted to find similarities and differences in the collected data [26]. For preliminary coding of the material, all transcripts were read and reread by the first author together with the author from Gauhati University. In the subsequent analysis, we focused on similarities and differences in the data in relation to the informants’ socioeconomic backgrounds. We defined preliminary categories and also compared the participants’ views with the dominant global discourses on surrogacy. Finally, in discussion with the other authors, we defined the final categories describing the key findings.

### Ethical considerations

Ethics approval was obtained from the Centre for Media Studies Institutional Review Board, New Delhi IRB (nr: IRB00006230). Prior to participating in the study, all participants received information about the purpose of the study, that measures would be taken to ensure confidentiality, that their participation was voluntary, and that they could withdraw at any time. Informed written or verbal consent was provided by all participants. Instructions were given to the interpreter and translator that they should keep all of the material confidential.

## Results

The themes found in the analysis were: *surrogate mother seen as prostitute*; *one’s ‘own child’ through IVF and surrogacy*; *commercializing motherhood*; and *surrogate mothers do a noble deed*.

Surrogacy was seen as an acceptable reproduction method as it provides a childless couple with their ‘own’ child with whom they share a genetic relation, in line with cultural expectations of parenthood. However, there were diverse views about the surrogate mother. Lack of knowledge about how surrogacy was conducted, that is to say, through IVF, made some view her as a prostitute. A view that the surrogate mother violated the concepts of motherhood through ‘selling her own child’ caused informants to judge her as a ‘bad woman.’ However, a contrasting view about the surrogate mother was presented by some informants who instead saw her as a woman worthy of respect for helping a childless couple.

Over the course of the 20 months during which the study took place, awareness and views of surrogacy seemed to change in Assam. Among the people encountered in spring 2012 at CSOs or people in general in Guwahati (mainly from the higher socioeconomic group), few knew much about surrogacy or else they did not want to talk about it. Surrogacy was described as a ‘hush-hush’ subject. However, by the end of 2013, people had become more outspoken on the issue. Although the awareness of surrogacy had increased in Assam, it was still described as an alien phenomenon and was treated as a delicate subject by all of those with whom we spoke.

During the study period, it was observed that there was an increase in the number of clinics offering the surrogacy procedure. In the spring of 2012, only two clinics could be found that provided this service, but, by the end of 2013, that number had doubled. Nevertheless, IVF doctors at these clinics said that it was difficult to find potential surrogate mothers. Women would reject the proposal on the grounds that they would not be able to be with their own children during the process because they had to live close to the clinic. Others said their husbands would not allow them to be surrogate mothers because of the risk of stigma.

In the four individual interviews with women from high socioeconomic background and the FGDs, we presented the vignette about a childless couple’s options for solving childlessness to see whether surrogacy would be brought up as an option. Surrogacy was mainly mentioned by people in higher socioeconomic groups, among teachers, and by some students. IVF (often referred to as ‘test-tube babies’) was proposed as a solution by everyone except the majority of those in lower socioeconomic groups. The most frequently suggested solutions among the latter were either some kind of medical treatment such as artificial insemination, or else adoption. When asked whether they had heard about a woman giving birth on behalf of a childless couple, most participants in the lower socioeconomic groups seldom associated it with a medical activity:

Yes, I have heard about it, and it is happening here, but not from another’s sperm and egg. However, when one woman becomes pregnant, another woman may ask her, ‘Give me this child.’ Then she may give her the child. But I have not heard about this other thing. (Interview no. 19, woman with low socioeconomic background)

### Surrogate mother seen as a prostitute

In lower socioeconomic groups, the concept of giving birth on behalf of a childless couple was often associated with a woman having sexual intercourse outside of marriage with the husband of the childless couple, and this form of surrogacy was seen as unacceptable:

Interviewer: Have you heard of any woman who has given birth to a baby for a couple who have no children?

Informant: I have heard of this, but only heard, not met – not in this area.

Interviewer: What do you think of that?

Informant: I think it would be bad if my husband were to get involved with another woman in order to have a child. But if a couple has a child and gives it away to a childless couple [by adoption], then it is OK. (Interview no. 13, woman with low socioeconomic background)

Some students asserted that surrogate mothers were viewed as prostitutes in society. In one FGD, they reported that the method contradicted the values of their society and their religion. However, when participants were given a more thorough explanation of the IVF surrogacy process, its acceptability changed:

Interviewer: If there is no sex involved, if the couple who are childless put together their egg and sperm, and then insert this inside the womb of the woman who will give birth to their child, what do you think about her?

Informant: Yes, putting together the egg and sperm and inserting them inside the uterus of another woman is a totally scientific process. In such a case, we have no objection, and it is not against our social system. (FGD no. 9, men with low socioeconomic background in a rural area)

### One’s ‘own child’ through IVF and surrogacy

In most groups, participants agreed that the woman is most often blamed for childlessness in their society, and, in that case, she risks being excluded from ceremonies and public activities. Others thought that it was God’s will whether they would be granted children. It was mentioned that, among Hindus, who believe in reincarnation, being childless is associated with some sort of bad deed that one has done in an earlier life, and as a result childless women are stigmatized.

Although adoption was often mentioned as a solution to childlessness, there was a view expressed that an adopted child was not ‘one’s own,’ and for this reason adoptive parents were said to run the risk of not being cared for by such a child in their old age. Some said there would be a lack of affection in a parent–child relationship arranged through adoption, stressing the importance of having a child who is a ‘blood’ relation. From this perspective, IVF was seen as a good option, in all socioeconomic groups, although adoption would be more common and more financially feasible for those in the lower socioeconomic group:

Man no. 5: After all, there are lots of problems with adoption, so it would be better if there were some scientific process for giving birth to a baby.

Man no. 4: One’s own child is always one’s own child, whether the baby is born by test-tube [IVF] or by any other process. (FGD no. 9, men with low socioeconomic background in a rural area)

Almost no one in the lower socioeconomic groups mentioned surrogacy; however, among the group of teachers, and in the higher socioeconomic group, surrogacy was brought up as a suitable option, as it would provide childless couples with biological children:

Through surrogacy, a mother can have her child, even if she has some health problems. Maybe she is not able to conceive or has other physical difficulties, like heart disease or something. Surrogacy is again a good choice because the couple will have their own children. (FGD no. 16, men with high socioeconomic backgrounds in an urban area)

When surrogacy was explained to the lower socioeconomic groups, the method was also seen as a procedure that would allow other people to believe that the parents had the child on their own, and not through adoption:

This [surrogacy] will only be known to the doctor, and to the husband and wife. So nobody will know about it and other family members will think that it is her own child. Only she and her husband will know what procedure they have used. (FGD no. 7, women with low socioeconomic backgrounds in an urban area)

However, not all participants stressed the importance of having a genetic connection to the child. Among some students and women with higher socioeconomic backgrounds, this was of minor importance, and adoption was preferred. Social issues were given as a reason by one woman: ‘So, according to me, adoption would be better [than surrogacy], because when a child comes and is unprotected, uncared for, left alone, we should save its life’ (individual interview no. 4, woman with high socioeconomic background). Some students expressed similar views:

Student no. 3: No, the first option is test-tube baby and secondly I rather go for proper adoption than for surrogacy, that is my personal opinion. Others may think differently, but for me it is not so important to have an own child so it is different for different people. (FGD no. 2, female students in an urban area)

### Commercializing motherhood

Recognition that there will be emotional bonding between the surrogate mother and child made many conclude that giving birth on behalf of someone else was unfeasible:

Woman no. 3: Everybody wants to bring up their child in their own home, even after facing the problems of survival [i.e. poverty]. So no one will ever give their child to another couple for money.

Interviewer: I understood that someone was trying to recruit women in this area but it was not possible to find any woman who was willing to do this. Why do you think this is?

Woman no. 3: Because after giving birth to a child a unique emotional attachment is created between the child and the mother. In such a situation, it is very difficult to give that child away to another couple. (FGD no. 10, women with low socioeconomic backgrounds in a rural area)

The factor of money being involved in the surrogacy process was considered by most informants in lower socioeconomic groups to be against the norms of their society:

People will say that she has sold off her baby. Selling a baby would not be tolerated in our society and would be considered a shameful behavior. People will not accept it. The [surrogate] mother will be called a bad [of degrading character] woman. (Interview no. 5, woman with low socioeconomic background)

The commercial appropriation of motherhood appalled many of our informants in this social group. In view of the emotional bond that they assumed between the surrogate mother and the child, they found the idea of giving away a child in exchange for money deeply disturbing:

Woman no. 3: We would not tolerate it! If a woman gives a baby to another couple just for money, it would be considered a bad practice – because if she does that, it would mean she prefers money to her own child.

Woman no. 4: She would be called a greedy woman.

Interpreter[explaining]: But the child is not from her own egg.

Women nos.3 and 4: Yes, we got it, but still people will have these opinions.

Women nos.2 and 5: Yes, they will call it a business. (FGD no. 8, women with low socioeconomic backgrounds in a rural area)

Some of the negative reactions concerning a monetary transaction were linked to religion:

Man no. 4: If this happens in a village and it becomes known to everyone that the woman is giving birth for money, then that woman may be socially ostracized from society.

Interviewer: Why would that be so?

Man no. 4: There are cultural and religious restrictions in our Hindu society that would never allow this. If someone does this, it will be called illegal and would not be acceptable. (FGD no. 9, men with low socioeconomic backgrounds in a rural area)

Giving birth was generally seen as involving such a strong emotional bond between mother and child that even poverty would not be a valid reason for giving the child away. However, different perceptions of how surrogate mothers would feel were expressed:

Woman no. 2: If the woman is a professional [working as a surrogate mother], then she will feel nothing. But if she is a common woman she may be emotionally disturbed for a long time – or even a lifetime – for giving her child away to another family.

Woman no. 1: I think she will certainly be emotionally disturbed, even if she is a professional. It is not a matter of whether she is paid or not, but whether she will feel emotionally disturbed for a long time. (FGD no. 4, female teachers in a semi-urban area)

### Surrogate mothers do a noble deed

Although the financial aspect of surrogacy created many negative reactions with regard to the surrogate mother, her actual deed – helping a childless couple – was seen by many of our informants as a selfless act. The view that the surrogate mother would risk emotional suffering caused some to regard the surrogate mother with great respect: ‘In society, it should be hats off to a surrogate mother. We should give her due respect for giving a beautiful gift. Society should respect that. I will respect her. The pain and trouble she is going through during pregnancy’ (individual interview no. 4, woman with a high socioeconomic background). Similar perceptions were found among both women and men, regardless of socioeconomic background, often pointing towards the great service done for others: ‘She has donated her baby and helped the childless couple, so she has done a good job and performed a noble deed’ (FGD no. 7, women with low socioeconomic backgrounds in an urban area).

Another woman in the same group pointed out the risk of conflict in the household of a childless couple, arguing that a surrogate mother would be of great help to such a family: ‘She is doing good, bringing peace to a family where there may be violence, so she is doing a good thing’ (FGD no. 7, women with low socioeconomic backgrounds in an urban area). In another group, in which a woman had commented earlier that this kind of ‘service’ would be difficult to perform, it was later agreed that this is an act deserving respect:

If someone can give her baby to another couple, we should thank her for performing such a noble deed. If she can sacrifice her child to make someone happy, we feel she is a good woman and has done something in the service of God. (FGD no. 10, women with low socioeconomic backgrounds in a rural area)

We have previously mentioned that some informants did not consider poverty a valid reason to be a surrogate mother. However, many assumed that poverty would be the reason for becoming a surrogate mother, and some even saw the emotional bond as a justification for the surrogate mother receiving compensation:

Many voices: We would accept her receiving a payment, since she has gone through all the pains of childbearing and has sacrificed her motherly love. We would never object to that because after carrying that child in her womb for nine months and then giving that baby to another she is entitled to the money. We would not be opposed to that. (FGD no. 11, men with low socioeconomic backgrounds in a rural area)

Student no. 4: It is not important how much money she gets. She is entitled to accept the money because she is sacrificing her labor and her motherly feelings. (FGD no. 12, male students in a rural area)

## Discussion

Assam is a state in India where few surrogacy clinics have been established. In our study we have shown that people seem to have little knowledge of surrogacy, particularly in lower socioeconomic groups. However, we observed a trend of an increasing number of clinics and awareness of surrogacy as a phenomenon in this community.

Although the informants did not know about the procedure beforehand, when the interpreter explained what the process involved, informants’ reasoning around this reproduction method showed that it fulfills the cultural expectations of parenthood and offers the much-wanted possibility of having one’s ‘own child.’ This view might also be related to the view that the sperm (which most often is contributed by the intended father) in Indian society is seen as the main provider of identity, while the egg (which is rarely supplied by the intended mother) is considered of minor importance [27]. If performed in secrecy, a childless couple may escape the shame of infertility and childlessness. It is, therefore, not surprising that surrogacy is on the rise as a route to parenthood for childless couples in India [10]. However, drawing from the narratives in this study, the surrogate mother might still be in a precarious situation, as her act is mainly considered reprehensible by many in the lower socioeconomic group. Accurate knowledge of IVF surrogacy is uncommon. When surrogacy is mentioned, it is often associated with sexual intercourse, an understanding that stigmatizes the surrogate mother, as other studies have also found [11,12]. Even when surrogacy is known not to involve intercourse, there is a risk that the surrogate mother may be stigmatized for a different reason: giving birth is strongly linked to marriage, but in the case of surrogacy, the birth-giving woman is not married to the child’s biological father [28].

The view of the surrogate mother is also intimately bound up with the concept of motherhood and the assumed emotional bonding of mother and child. The belief that the surrogate mother is giving away her ‘own child’ appears to have profound effects on how her actions are perceived. The belief of an emotional bond also came with a notion of emotional suffering from giving up the motherhood role, and this resulted in an empathetic view of the surrogate mother. However, when the element of money is added, for many in the lower socioeconomic groups it conflicts with the construction of the woman as a mother. This is more strongly reacted against by women, who might have experienced giving birth and feelings of an emotional bond towards the child, strongly linked to motherhood. With the involvement of money, surrogacy is understood as more of an economic transaction than a motherly act. Motherhood becomes commercialized, which is contrary to the values of society in Assam. However, a contrasting view among informants places the surrogate mother in a praiseworthy position.

Although our informants acknowledged the risk of emotional suffering, the notion of exploitation was not directly noticeable, as it is in the main global discourse [1–5]. Those with whom we spoke did not mention that an impoverished woman might be ‘forced’ into surrogacy, as the exploitation discourse declares. Because many of our informants had little or no understanding of surrogacy, their perceptions might have been different if they had known about the process in the surrogacy clinics.

Today, conflicting discourses on exploitation and empowerment are the most prominent perspectives on surrogacy and affect judgments related to it. It has been suggested that the exploitation discourse originated among Western scholars, in which case, ‘we must listen very carefully to Indian women’s voices, and be mindful of the possibility that Western theoretical tools may have harmful effects when exported’ ([29],p.726).

Indian researchers argue that if surrogacy were to be banned in India it would be disadvantageous to the surrogate mothers who would lose the possibility of earning a significant amount of money. Instead, they contend that it should be better regulated and especially provide protection for the surrogate mother [30,31]. However, local understandings of surrogacy also need to be taken into account to further be able to protect the surrogate mothers. For example, both the exploitation discourse and the empowerment discourse view the surrogate mother as a vulnerable woman who may either be harmed or benefitted by the surrogacy arrangement. However, neither of these discourses describes a surrogate mother as many of our informants did: as someone violating the norms of motherhood. Any efforts to protect surrogate mothers by legislation should consider the potential social consequences of the surrogate mother’s actions within the community where she lives.

Policy makers should be mindful of the local views of motherhood and how they may contrast with the discourse at surrogacy clinics, where the surrogate mother is only seen as someone carrying another person’s child [32]. Motherhood in an Indian context is linked to the woman giving birth [27,32], and hence, in our study, the surrogate mother is viewed by many as commercializing motherhood. This, in addition to the belief that sexual intercourse is involved, leaves the surrogate mother stigmatized. Even if surrogate mothers earn a great deal of money, they risk ostracism and in the end may be worse off for their efforts.

Policies and laws can stigmatize a practice, depending on how they are framed [33]. The view that surrogate mothers are doing a noble deed corresponds with Amrita Pande’s suggestion that surrogacy should be seen as ‘care work’ [11]. Referring to it as such, rather than as ‘surrogate motherhood,’ might also counteract the view that surrogacy has made motherhood a commercial undertaking. With the act seen as work instead of motherhood, while still recognizing an emotional factor in carrying a child, both the local view of emotional bonding and the fact that childbearing is being conducted for someone else are taken into account.

### Methodological considerations

Most of our participants from lower socioeconomic groups were not influenced by any previous knowledge of surrogacy, and none of the informants gave reason to believe that they were influenced by the global discourses on surrogacy. It is a strength of this study that opinions were gathered in a region where this phenomenon is still new, and before most participants had been affected by any positive or negative accounts of surrogacy in the media. However, the lack of previous knowledge of surrogacy is also a limitation of the study. The informants were asked to give immediate responses about an unknown reproduction method, explained by the interviewer in the very moment of the interview. Nonetheless, the method used in this study made it possible to capture uninfluenced perceptions. There was admittedly the risk of the interviewer influencing the informants by the way of presenting the reproduction method. Further, the informants might have changed their opinions after some reflection, given that this method was difficult to understand for some participants. Still, even though we must be careful with what conclusions we draw from this study, the informants’ narratives on surrogacy show how this reproduction method both fulfils and contrasts with some sociocultural expectations of parenthood and particularly motherhood.

The range of different socioeconomic groups in our study and the use of both individual interviews and FGDs enhanced the credibility of the findings. Although we did have difficulties in arranging a FGD with women from a higher socioeconomic background, we were able to obtain a few individual interviews from this group in society. Additionally, the first author gained more insight into the awareness and perceptions of surrogacy in this context by spending time in Assam. The second author, working in Assam, could also verify the meaning of the narratives, which helped to increase the trustworthiness of the analysis.

The difficulties of using an interpreter, especially in the FGDs where important issues might have been missed for probing, can also be seen as a limitation. However, our interpreter did do some probing, which limited the risk of missing important follow-up questions.

Since India is socially and culturally very diverse regarding for example religion and caste, it is a limitation that only a few Muslims (two women and three men) were individually interviewed, as they might have had different opinions than Hindus. Still, the views of the Muslims in our study did not seem to differ from those of the Hindus.

## Conclusion

The view that the surrogate mother is someone doing a noble deed is supported by the notion that ‘care work’ is being done in surrogacy. Thus, in the interaction between the surrogate mother and the intended parents, a surrogate mother would ideally be someone who is valued and respected, as well as financially compensated, for her services. Although economic conditions may constrain an impoverished woman’s choices, once she has entered into a surrogacy agreement, her rights may be secured by proper regulations. On the other hand, when regulating this reproduction method, one cannot ignore that there might also be the risk of emotional suffering for the surrogate mother.

If surrogacy is seen as ‘care work,’ the rights of surrogate mothers can be incorporated into existing labor rights legislation, as has been suggested by Pande [30]. Such regulations may be modified to take into account local norms, thereby reducing the possibility of stigma and other forms of social discrimination towards surrogate mothers.

### Further research

Findings from our study, in line with what has been shown in other studies, reveal that a genetically related child is of great importance in Indian society. However, among informants in Assam, there seemed to be some contrasting views among the higher educated in this community. The findings suggest that the higher the level of education, the less value is placed on the importance of a child of one’s ‘own’ blood, and that adoption is preferred before surrogacy as a solution to childlessness. Further research is needed to understand how societal views regarding parenthood are changing as a result of education and upward socioeconomic mobility.

## Paper context

Commercial surrogacy has been allowed since 2002 in India, but it is still not regulated. Regulations have been called for to protect surrogate mothers and counteract exploitation. There are scarcely any studies on the general population’s perceptions of surrogacy. It is important to gain insights into local understandings of this reproduction method before such regulations are formulated. This study serves to provide local understandings from Assam, a northeastern state in India, about surrogacy and surrogate mothers.
